# A pragmatic randomized controlled trial of a group self-management support program versus treatment-as-usual for anxiety disorders: study protocol

**DOI:** 10.1186/s12888-021-03675-4

**Published:** 2022-02-21

**Authors:** Pasquale Roberge, Janie Houle, Jean-Rémy Provost, Simon Coulombe, Annie Beaudin, Peter Bower, Félix Camirand Lemyre, Martin Drapeau, Marc-Simon Drouin, Catherine Hudon, Martin D. Provencher, Helen-Maria Vasiliadis

**Affiliations:** 1 Department of Family Medicine and Emergency Medicine, Faculty of Medicine and Health Sciences, Centre de recherche du Centre Hospitalier Universitaire de Sherbrooke (CRCHUS), Université de Sherbrooke, 3001, 12th Avenue North, Sherbrooke (Quebec), J1H 5N4 Canada; 2 Department of Psychology, Faculty of Social Sciences, Université du Québec à Montréal, C.P. 8888, succ. Centre-ville, Montréal (Quebec), H3C 3P8 Canada; 3 Relief, 418, rue Sherbrooke Est, bur. 300, Montréal (Québec), H2L 1J6 Canada; 4 Département des Relations Industrielles, Université Laval, Pavillon J.-A.-DeSève, 1025, avenue des Sciences-Humaines, Québec, G1V 0A6 Canada; 5 VITAM – Centre de recherche en santé durable, Québec, Canada; 6 National Institute of Health Research School for Primary Care Research, The University of Manchester, Manchester, M13 9PL UK; 7 Department of Mathematics, Faculty of Sciences, Centre de recherche du Centre hospitalier universitaire de Sherbrooke (CRCHUS), Université de Sherbrooke, 2500, boul. de l’Université, Sherbrooke (Quebec), J1K 2R1 Canada; 8 Departments of counselling psychology and psychiatry, McGill University, 3700 McTavish, Montreal, Quebec, H3A 1Y2 UK; 9 School of Psychology, Faculty of Social Sciences, Université Laval, 2325, rue des Bibliothèques, Québec, G1V 0A6 Canada; 10 Centre de recherche Charles-Le Moyne, Department of Community Health Sciences, Faculty of Medicine and Health Sciences, Université de Sherbrooke, 3001, 12th Avenue North, Sherbrooke (Quebec), J1H 5N4 Canada

**Keywords:** Anxiety disorders, Self-management support, Pragmatic trial, Group intervention, Web-based intervention, Transdiagnostic

## Abstract

**Background:**

The integration of a personal recovery-oriented practice in mental health services is an emerging principle in policy planning. Self-management support (SMS) is an intervention promoting recovery that aims at educating patients on the nature of their mental disorder, improving their strategies to manage their day-to-day symptoms, fostering self-efficacy and empowerment, preventing relapse, and promoting well-being. While SMS is well established for chronic physical conditions, there is a lack of evidence to support the implementation of structured SMS programs for common mental disorders, and particularly for anxiety disorders. This study aims to examine the effectiveness of a group-based self-management support program for anxiety disorders as an add-on to treatment-as-usual in community-based care settings.

**Methods/design:**

We will conduct a multicentre pragmatic randomized controlled trial with a pre-treatment, post-treatment (4-month post-randomization), and follow-ups at 8, 12 and 24-months.

**Treatment and control groups:**

a) group self-management support (10 weekly 2.5-h group web-based sessions with 10–15 patients with two trained facilitators); b) treatment-as-usual. Participants will include adults meeting DSM-5 criteria for Panic Disorder, Agoraphobia, Social Anxiety Disorder, and/or Generalized Anxiety Disorder. The primary outcome measure will be the *Beck Anxiety Inventory*; secondary outcome measures will comprise self-reported instruments for anxiety and depressive symptoms, recovery, self-management, quality of life, and service utilisation.

**Statistical analysis:**

Data will be analysed based on intention-to-treat with a mixed effects regression model accounting for between and within-subject variations in the effects of the intervention.

**Discussion:**

This study will contribute to the limited knowledge base regarding the effectiveness of structured group self-management support for anxiety disorders. It is expected that changes in patients’ self-management behaviour will lead to better anxiety management and, consequently, to improved patient outcomes.

**Trial registration:**

ClinicalTrials.gov: NCT05124639. Prospectively registered 18 November 2021.

## Background

### Background and rationale

Anxiety disorders are prevalent and disabling mental disorders characterized by marked fear, anxiety and avoidance behavior [1–3]. More frequent in women than men, they often appear during childhood and adolescence, and 50–80% of cases are comorbid with other anxiety disorders, mood disorders, and substance use disorders [4–7]. They also frequently coexist with chronic physical illness [8]. Individuals with anxiety disorders demonstrate significant psychological distress, functional and social impairment, suicide risk, and service utilization [1, 9–13]. The Global Burden of Disease Study ranks anxiety disorders as the sixth leading cause of years of life lived with disability [14]. Pharmacological and psychological treatments are recommended in clinical practice guidelines for the management of anxiety disorders [1, 15]. However, anxiety disorders often present a relapsing or chronic course [16–20], and residual symptoms are frequent, even among patients in remission [21, 22]. Thus, individuals have an active role to play in their lifelong recovery, beyond the contribution of evidence-based treatments, to develop self-management skills and improve functioning, prevent relapse and live a fulfilling life despite the presence of residual symptoms [23, 24].

Self-management support (SMS) is an intervention promoting recovery that aims at educating patients on the nature of their mental disorder, improving their strategies to manage their day-to-day symptoms, fostering self-efficacy and empowerment, preventing relapse and promoting well-being [25–27]. SMS is consistent with patient-centered care [28] and has the potential to enhance the efficiency of the health care system for patients by improving health outcomes and reducing overall service utilization [29]. SMS is a promising avenue towards recovery by fostering social inclusion, self-determination, autonomy, hope, and personal responsibility. Recovery is defined by the Mental Health Commission of Canada [30] as “living a satisfying, hopeful and contributing life, even when there are ongoing limitations caused by mental health problems and illnesses”. Previous studies have found that patients in recovery from anxiety disorders use a large variety of self-management strategies in their day-to-day life to foster their recovery [24, 31]. No less than 60 different self-management strategies have been identified in a qualitative study among patients in recovery from mood or anxiety disorders [31]. It is expected that changes in patients’ self-management behaviour will lead to better anxiety management and, consequently, to improved patient outcomes, relapse prevention, lower utilization of health care services, and cost savings. SMS typically offers a wide variety of self-management strategies that each person can draw upon based on their needs and preferences to make informed health decisions in their everyday lives, with a predominant focus on self-efficacy, active engagement in the long term, relapse prevention, peer support and facilitation approach. SMS interventions are positioned as a complementary intervention to evidence-based pharmacological or psychological treatments for anxiety disorders and offer a specific contribution to interdisciplinary mental health practice. While some overlap (e.g., psychoeducation, problem solving, relapse prevention) with low-intensity cognitive behavioural therapy (CBT) is acknowledged, a primary difference is that structured SMS programs tend to implement a holistic recovery-oriented approach centered on optimizing wellness and living a fulfilling life even in the presence of ongoing symptoms, to empower people to develop their very own self-management toolbox in a supportive framework, and to be provided by people with a large diversity of backgrounds (e.g., health care providers, social and community workers, peer supporters) [25].

While the value of the integration of SMS programs to health care services for chronic physical conditions is well-established [27, 29, 32] and rapidly growing for depression [25, 33–36], few studies have examined the added value of structured SMS programs for anxiety disorders as a complement to usual care. In a review by Houle et al. [25], the efficacy of SMS for depression was examined in six studies and promising results were observed for symptom reduction, self-management behaviours and self-efficacy, and mixed results for relapse rates. A recent literature search identified two other studies, conducted in the United States, that evaluated the efficacy of SMS group interventions, that were both peer-led and administered to heterogeneous samples of patients with mental health problems [34, 37]. While promising, most studies did not examine patient outcomes for anxiety disorders. Only two trials were found specifically for self-management support in anxiety disorders. In Germany, a cluster-randomised controlled trial of a nurse-led collaborative care intervention to promote self-management support has shown a small effect on self-efficacy in primary care patients with anxiety, depressive or somatic symptoms [38]. In the Netherlands, a randomized controlled trial evaluated a group rehabilitation and self-management program among patients with chronic anxiety and/or depression in an outpatient mental health care setting and reported a moderate effect on empowerment, but did not observe any significant effects on quality of life or symptom severity [39]. Consequently, there is a knowledge gap about the added value of structured SMS programs to usual care for common mental disorders, and particularly for anxiety disorders.

A program of self-management workshops for mental health has gathered interest from policy makers, health care managers, clinicians, and patients alike over the past few years. The *J’avance!* program was developed by a well-established mental health community-based organisation in Quebec, Relief (https://monrelief.ca), whose mission is to help individuals with anxiety disorders, depression, and bipolar disorder. The SMS program designed for anxiety disorders draws on personal recovery models as well as on low-intensity psychosocial and psychological interventions; it has been thoroughly developed in collaboration with researchers (JH; scientific lead) and reviewed by an interdisciplinary expert committee as well as participants from pilot groups. The SMS program covers a broad range of mental health intervention strategies (e.g., problem solving, emotion regulation, exposure, cognitive restructuring, mindfulness) and wellness-focused approaches (e.g., strengths, social support, lifestyle habits) in a non-directive “toolkit” aimed at building self-management skills. Great emphasis is placed on peer support, with participants sharing experiential knowledge, committing to trying self-management strategies and overcoming stigmatization. Since 2014, the SMS intervention was delivered over 445 times and over 1000 facilitators have been trained to date, and implementation is also beginning across Canada and internationally. Initially an on-site only workshop, Relief has implemented in 2020 a virtual delivery format on an eLearning platform and is now conducting group SMS workshops with both modalities. Given this wide-spread implementation, we sought to examine how the group SMS workshop translates into better mental health outcomes, health care system utilization and overall efficiency as a complement to usual care for patients with anxiety disorders.

### Objectives

The aim of the present study is to evaluate the effectiveness of a structured group virtual SMS program as an add-on to treatment-as-usual (TAU) in a sample of adults with anxiety disorders. Primary questions: When group SMS is added to TAU in community-based care for patients with anxiety disorders, is the SMS + TAU group more effective in reducing anxiety symptoms than TAU alone? Secondary questions: a) Considering a recovery-oriented approach for patients with anxiety disorders, is there a significant difference between group SMS + TAU and usual care in terms of self-management strategies and personal recovery assessment at the 12-month follow-up? b) Does group SMS + TAU present superior cost-effectiveness and cost-utility, in terms of quality of life and anxiety-free days, than TAU for patients with anxiety disorders at the 12-months follow-up? c) Is there a significant difference between group SMS + TAU and usual care for high-end functioning rates? d) Is there maintenance of gains at 12- and 24-months follow-up? e) Is there differential effectiveness based on moderators (i.e., sociodemographic characteristics, clinical characteristics, past treatment experience) and mediators (i.e., group cohesion, therapeutic alliance, adherence)?

### Trial design

The trial is a two-arm parallel group multicentre pragmatic superiority randomized controlled trial (RCT), with a 1:1 allocation at the individual level. The group SMS intervention will be offered to participants in the TAU groups after the 12-month follow-up (delayed-intervention). The 24-month follow-up will therefore only provide a within-group dataset. The proposed protocol conforms to SPIRIT guidance [40].

## Methods

### Participants, interventions, and outcomes

#### Study setting

The study will be conducted in four health administrative regions in Quebec (Canada): Eastern townships, Mauricie-et-Centre-du-Québec, Abitibi-Témiscamingue and Laurentides. Administrative regions were purposefully selected based on the following criteria: a) the in-person group SMS intervention is not currently largely implemented in the region; b) the virtual SMS intervention is rarely accessed by participants from the region, even though the virtual SMS format is technically accessible throughout the province; c) diversity (e.g., population size, region, university teaching hospital).

#### Eligibility criteria

This pragmatic RCT focuses on broad inclusion criteria for mixed anxiety disorders groups and minimal exclusion criteria. *Inclusion criteria:* (1) aged 18 and over, (2) fluent in spoken and written French, (3) meeting DSM-5 diagnostic criteria for at least one of the following anxiety disorders: Panic Disorder, Agoraphobia, Generalized Anxiety Disorder, and Social Anxiety Disorder, (4) access to a computer or tablet connected to the internet with microphone and video camera. *Exclusion criteria:* (1) previous enrolment in the SMS intervention for anxiety disorders provided by Relief, (2) active suicidal intentions, (3) severe depressive symptoms (i.e., PHQ-9 score ≥ 20), (4) active substance-related and addictive disorder, and (5) cognitive impairment.

#### Recruitment

**Fig. 1:**
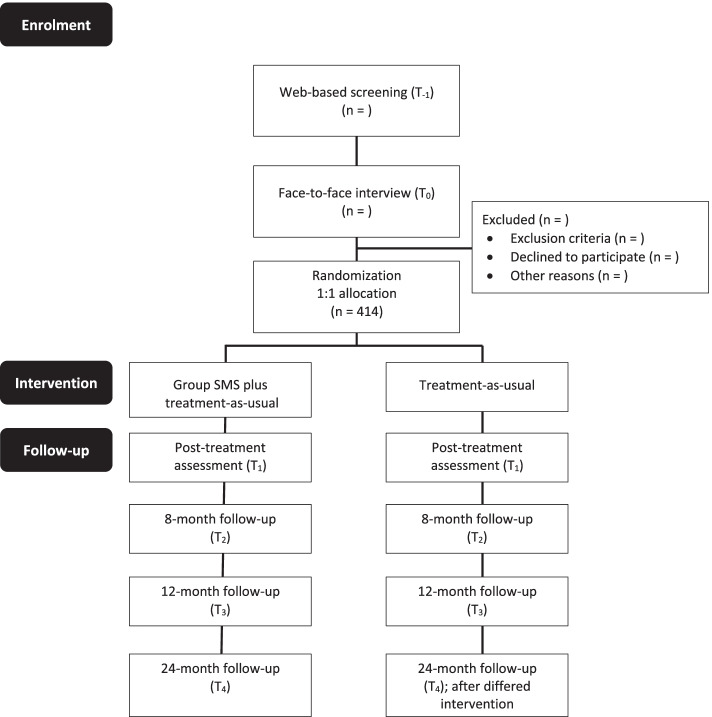
Flow of participants

Recruitment strategies will include self-referral following advertisements (e.g., waiting rooms of clinics, bulletin boards, geo-located website, and social media) and referrals from community-based primary care (e.g., family physician, community organization, mental health care team, mental health provider). The recruitment of participants will be conducted through a two-stage process. *Filter 1:* Self-referred individuals will acquire information on the study by accessing the study’s website or through a telephone call or email to our research laboratory. Self-referred individuals will complete a web-based screening survey comprising the required online consent form, basic eligibility criteria as well as anxiety symptoms and comorbidity overview. The initial web-based consent form and procedure has been approved by the ethics committee. At the end of the survey, they will provide their name and contact information. In the presence of clear exclusion criteria, a list of mental health resources will be provided. *Filter 2:* In the second stage (within 2 weeks of the screening survey), the baseline assessment will be conducted on a secure web-based platform with a trained clinical evaluator. The interview will begin with the consent form. The evaluator will explain the study, review the consent form with the participants, answer their questions, and verbally ask for their consent. The consent form will be sent by email for the participants to read prior to the online assessment, and this verbal consent procedure has been approved by the ethics committee given the web-based data collection method. The assessment will comprise sociodemographic data, service utilization and MINI International Neuropsychiatric Interview [41] for DSM-5 assessment (T_0_; random pre-assignment), combined with an adapted baseline Relief interview procedure (e.g., current main difficulties, interest in the program, capacity to use eLearning technology, readiness to take part in a group intervention). Patients meeting eligibility criteria at T_0_ will be given instructions to complete the remaining web-based self-reported questionnaires within 48 h, and only then will we have all required information to proceed with randomization. Figure 1 shows the study flowchart.

#### Interventions

##### Group SMS for anxiety disorders + treatment-as-usual (TAU)

The SMS manualized program for anxiety disorders (https://monrelief.ca) aims at improving self-management capabilities through weekly 2.5-h sessions with 10–15 patients over a 10-week period. The SMS program (see Table 1) covers the following themes: getting to know your anxiety; building self-awareness; reconsidering your lifestyle habits; adopting a problem-solving method; avoidance and exposure; acceptance and committed action; seeing things differently; managing your emotions; receiving support from others; and consolidating your toolkit. The trial will focus solely on the virtual format. As in the groups delivered by Relief, SMS will be either co-facilitated by health care professionals (e.g., social worker, psycho-educator, nurse), or by a health care professional and a peer supporter, namely a person recovered from an anxiety disorder who has experience and training offering peer support, building on mutual understanding and respect [42–44], to emphasize experiential knowledge sharing. *Relief* will provide the material for the training of the facilitators, the program documentation, the eLearning platform, and the material used by the participants. Consistent with standard implementation of this SMS program, facilitators will participate in a one-day *Relief* training program. Three case discussions for each group delivered (before onset, mid-group, and following last session) will be conducted by the research team. A random review of 30% of recordings of sessions will be conducted to monitor adherence with a treatment integrity scale. Integrity data will not be used to intervene to improve compliance, but only to examine process-outcome correlation and to guide improvements following the trial. Patient compliance will be supported through the extensive Relief experience delivering SMS (e.g., material, training, support), and the convenient eLearning platform (e.g., easy access, reduced stigma).

**Table 1:** Content of the 10-week SMS manualized group program for anxiety disorders

Week	Theme	Content
1	Getting to know your anxiety	• Learn more about anxiety • Recognize the signs of anxiety
2	Building self-awareness	• Develop self-observation skills to better understand your anxiety • Understand the function of different resources that are available
3	Reconsidering your lifestyle habits	• Understand how lifestyle habits influence anxiety • Use an action plan to initiate change • Learn about mindfulness • Incorporate relaxation techniques
4	Adopting a problem-solving method	• Learn to put a problem-solving strategy into practice
5	Avoidance and exposure	• Understand how avoidance works • Learn the exposure technique
6	Acceptance and committed action	• Understand the acceptance process • Take action despite anxiety
7	Seeing things differently	• Become aware of filters (cognitive distortions) • Recognize and mitigate the negative effects filters have on anxiety
8	Managing your emotions	• Decipher the messages your emotions are sending • Become better equipped to manage your emotions
9	Receiving support from others	• Realize how anxiety influences interpersonal relationships • Appreciate the importance of social support • Expand your support network
10	Consolidating your tool kit	• Acknowledge your progress • Recognize and consolidate your new skills

##### Treatment-as-usual

No limitations will be imposed concerning usual care, as we aim at examining the added value of SMS to TAU. To reflect heterogeneity of health seeking behaviour and mental health practices for anxiety disorders in the community, we do not require that participants have a family physician, be constrained to a prespecified usual care or have contacts with the healthcare system. To minimize behavioural change in healthcare providers, we will not inform any healthcare provider of participation in the study. As the intervention will be provided on an eLearning platform, and not embedded in clinics, contact between intervention and control patients is unlikely. All participants will receive information about the SMS intervention and the goal of the study, i.e., “of helping them manage their anxiety”. We will thoroughly assess participant-reported service utilization 12 months prior to enrolment and during the study to examine risk of study-induced behavioral change with regards to usual care.

#### Participant assessment

Table 2 shows the assessment timeline. The data collection will be based on instruments with good psychometric properties, previously used in clinical trials for anxiety disorders to ensure comparability, and with validated French versions (when available).

**Table 2:** Study schedule of patient assessment

TIMEPOINT	Web-based screening	Web-based interview and questionnaires-Enrolment	Intervention				
			Follow-up				
	T_**−1**_	T_**0**_		T_**1**_	T_**2**_	T_**3**_	T_**4**_
**INITIAL SOCIODEMOGRAPHIC AND CLINICAL ASSESSMENT**							
Sociodemographic variables	X	X					
Social Phobia Inventory	X						
Panic Disorder Severity Scale	X						
Mini International Neuropsychiatric Interview (MINI)		X					
Disease Burden Morbidity Assessment		X					
Service utilization and medication (past 12 months)		X					
**SYMPTOM-FOCUSED OUTCOMES**							
Generalised Anxiety Disorder – 7	X			X	X	X	X
Beck Anxiety Inventory		X		X	X	X	X
Patient Health Questionnaire – 9	X			X		X	X
**RECOVERY-FOCUSED OUTCOMES**							
Recovery Assessment Scale - Revised		X		X		X	X
Mental Health Self-Management Questionnaire		X		X		X	X
**ECONOMIC EVALUATION OUTCOMES**							
AQoL-6D		X		X	X	X	X
Service utilization and medication (past 4-months)		X		X	X	X	X
**PROCESS MEASURES**							
Working Alliance Inventory			X				
Gross Cohesion Scale			X				
Logbook			X				
**EXPERIENCE WITH THE INTERVENTION**							
Brief qualitative interview						X	X

##### Baseline

Sociodemographic variables will be collected at baseline (T_−1_ et T_0_), and comprise sex and gender, age, marital status, racial identity, ethnicity, education level, income level, occupation, and insurance coverage. We will also collect data on previous experience with mental health services. The web-based screening survey will comprise the Generalised Anxiety Disorder-7 (GAD-7) [45], a 7-item self-report questionnaire measuring anxiety symptomatology. Diagnostic-specific measures will also be administered. The Social Phobia Inventory (SPIN) [46, 47] is a 17-item self-report questionnaire measuring the fear, avoidance, and physiological discomfort associated with social anxiety disorder. Studies have reported good internal reliability, test-retest reliability, and convergent validity [46]. The Panic Disorder Severity Scale Self Report (PDSS-SR) [48] is a questionnaire measuring the severity of seven dimensions of panic disorder. The PDSS-SR shows good internal reliability, test-retest reliability and sensitivity to change [48]. The clinical assessment will be based on the Mini International Neuropsychiatric Interview (MINI) [41], a brief structured diagnostic interview for DSM-5 administered by a trained lay interviewer. Inter-rater reliability will be assessed for 25% of audio-recorded interviews.

##### Primary outcome measure

The severity of anxiety symptoms will be assessed using the self-report, 21-item Beck Anxiety Inventory (BAI) [49, 50]. The BAI assesses emotional, physiological, and cognitive symptoms of anxiety and indicates minimal (0–7), mild (8–15), moderate (16–25) and severe anxiety (26–63). The scale shows significant reliable improvement and clinically significant change cut-points [49–51].

##### Secondary outcome measures

Participants will also complete diagnostic-specific measures, as well as other questionnaires related to quality of life, self-management, and recovery. The Patient Health Questionnaire (PHQ-9) [52] is a 9-item, self -report questionnaire measuring the frequency of depressive symptoms with good reliability and validity. The Assessment of Quality of Life – 6D (AQol-6D) [53] is a valid and reliable 20-item questionnaire assessing six psychosocial and physical dimensions related to the quality of life. The Disease Burden Morbidity Assessment [54, 55] is a self-report questionnaire measuring the presence of chronic conditions and interference on daily activities. The self-administered Recovery Assessment Scale – revised (RAS-r) [56–59] is a 24-item validated patient-oriented outcome measure of recovery in five domains: personal confidence and hope, willingness to ask for help, goal and success orientation, reliance on others, no domination by symptoms. The RAS is the most frequently used and tested recovery measure, has good psychometric properties, including sensitivity to change, and correlates with a range of other measures (e.g., activation, psychological well-being, positive illness outlook). The Mental Health Self-Management Questionnaire (MHSQ) [23] assesses the use of mental health self-management strategies. It comprises 18 items. The scale has satisfactory internal reliability and construct validity, adequate test–retest reliability and its convergent and concurrent validity are supported.

##### Service utilization

Data will be obtained from provincial administrative databases (i.e., *Régie de l’assurance-maladie du Québec* (RAMQ), Quebec emergency department database (BDCU) and public primary health care database (I-CLSC)) for medical and biopsychosocial services, hospitalization’s registry, and medication data at the end of the data collection. A brief questionnaire on other mental health consultations (e.g., type of professional, duration, costs) and psychotropic medication will be administered at each assessment period to offset the limitations of administrative data.

##### Questionnaires completed during SMS sessions

The appreciation of the alliance for both participants and facilitators will be assessed with the 12-item version of the Working Alliance Inventory (WAI) [60, 61]. The WAI shows good construct validity and high internal consistency [62]. The perceived cohesiveness and bond for participants will be examined with the 9-item Gross Cohesion Scale (GCS) [63], a scale with acceptable reliability and validity. These measures will be used at sessions 3 and 8.

A logbook will also be used by facilitators to record intervention adherence for each participant as well as to report experiences and perceptions related to SMS group facilitation. The logbook content will provide a better understanding of the actual implementation of the program. The facilitators will also complete a brief questionnaire comprising sociodemographic questions, items on academic and professional backgrounds, as well as experience with SMS, group interventions and anxiety disorders.

### Embedded qualitative interview

A sequential embedded qualitative approach [64] will be used to explore participants’ views and experiences regarding the SMS intervention. The data collection will include a brief individual telephone contact with open-ended questions at the T_3_ and T_4_ follow-ups. A semi-structured interview guide [65] with open-ended questions will be used to elicit information on topics such as participants’ experience with the intervention, its perceived effectiveness, and most useful strategies or skills acquired. We will obtain verbatim transcripts of all the audio recordings. Data coding and analysis will be conducted based on the interactive cyclical process of data reduction, data display and conclusion drawing and verification [66]. For data reduction, we will use the QSR*NVIVO database software [67] to analyze the transcriptions with a coding strategy based on emerging clustering during the process.

### Data collection, management, and analysis

#### Participant timeline

The assessments will be conducted at baseline (T_−1_, T_0_), posttreatment (T_1_; 4-month post-randomization) and follow up at 8-month (T_2_), 12-month (T_3_) and 24-month (T_4_) post-randomization. In-treatment assessments for participants and facilitators will also be conducted at sessions 3 and 8. Self-report questionnaires at each assessment period will be completed online through the REDCap application managed at the *Centre de recherche du Centre Hospitalier Universitaire de Sherbrooke* (CRCHUS) at University of Sherbrooke on a secure server with systematic backups. The clinical interviews (T_0_, T_3_, T_4_ only) will be conducted with the Zoom video conferencing service. Data collection with the REDCap application will be managed independently of the treatment assignment. The database will only include coded, depersonalised data, and participant’s identifying information will be stored in a separate secure location with restricted access to the linking code.

We conservatively planned for a 25% attrition at follow up, but we will devote considerable efforts toward a < 5% target with study retention strategies: (1) limiting burden and inconvenience (minimal data collection; web-based; secondary direct data capture through administrative databases); (2) minimal dataset for participants identified at high-risk of attrition (BAI, AQoL-6D); (3) monitoring data collection in real time; (4) education strategies on patient engagement and appreciation (e.g. reminders, website), (5) contact of dropouts; (6) information gathering for relocation; (7) financial compensation (20 $ for each follow up assessment); (8) treatment incentives with delayed-access to SMS for control arm to increase perceived health benefits. Follow up measures at 12- and 24-month are more susceptible to attrition (secondary analysis), and we will include a brief web-based interview that will foster patient engagement. Moreover, we will obtain a complete dataset from provincial administrative data on service utilization.

#### Assignment of interventions: Sequence generation, allocation concealment mechanism and implementation

Participants will be randomized based on stratification for study site, with random block sizes (2, 4, 6) to ensure a balance in the allocation for the strata. The randomization schema will be carried out using a code generated by the study statistician. Concealment will be maintained for the participants, research team, and staff. The REDCap computerized platform will only release the randomization code to the research coordinator based on the allocation sequence after verification of eligibility. The research coordinator will then enrol and assign participants to interventions.

The randomization sequence will be recorded with random codes (“A” and “B”) until the primary outcome analyses are concluded. Masking of trial participants and facilitators is not possible in this trial. Clinical evaluations at T_3_ and T_4_ will not be masked as we will address through qualitative interviews patient experience following the SMS intervention, or readiness to enroll intervention at T_3_ for the control group.

#### Sample size

Due to challenges in calculations for mixed regression models [68], we estimated sample size based on the baseline (T_0_) and post-treatment (T_1_) difference between groups for the primary outcome. The sample size was calculated using G*Power with the BAI based on an estimated effect size of the SMS + TAU intervention (Cohen’s *d*) of 0.32 (0.56 intra-group). This corresponds to a 7-point difference based on previous studies [51] that have established *clinically significant change* thresholds for the BAI [69]; such an effect size at post-treatment for symptom reduction is consistent with previous SMS studies for mental disorders [25, 35, 37, 38] as well as with pilot data of SMS (conservative estimates). The 3-point difference and SD for the TAU only group are conservative values based on our current data in a similar study with a TAU group [70]. A sample size of 155 individuals per group is therefore required to detect a 4-point (pooled SD: 12.5) pre-post treatment difference between SMS + TAU and TAU (i.e., a 7-point difference for SMS + TAU and 3-point for TAU) with an 80% power and α = 0.05. As there is a potential for intraclass correlations (ICC) in an individually randomized group treatment trial [71], we also estimated [72, 73] that based on a mixed model accounting for possible between-individual correlations within each intervention group (ICC of 0.02 for based on a similar study [70]), the power would be of at least 76%. With an adjustment for a conservative 25% attrition rate, the proposed sample size is 207 individuals for each treatment arm.

#### Statistical Analyses

##### Clinical outcomes

Statistical analysis will follow intent-to-treat principles. The primary outcome analysis will be performed at T_1_. The remaining analysis will be conducted considering all measures over time when all participants have completed the 12-month (T_0_ through T_3_) and 24-month follow up (T_0_ through T_4_). Primary question: A mixed model regression with the maximum-likelihood method will be performed to consider between- and within-subject variations in the analysis of the longitudinal effects of SMS + TAU compared to TAU on the primary outcome measure (BAI) at post-treatment (T_1_). To control for potential intra-group variability, random effects will be added on the participants nested in the SMS groups. Baseline clinical variables (e.g., anxiety disorders, comorbid depressive symptoms, psychotropic medication, psychotherapy) will be entered in the model as covariates. Analyses will be conducted with all available data without imputation, as estimation of parameters by maximum likelihood is considered adequate to address missing data as post-treatment in the multilevel model [74, 75].

For secondary questions, logistic regression models will be used to examine high-end functioning rates. The mixed model regression approach will be repeated on all data at the 12-month (T_0_ through T_3_) and 24-month (T_0_ through T_4_) follow-up, and will allow for the inclusion of patients with missing data at any of the follow-up assessments (T_1_,T_2_,T_3_,T_4_). Additionally, treatment effect sizes will be estimated with Cohen’s *d*. We will also conduct hypothesis-generating moderator and mediator analysis [76]. Moderation analyses will be performed for three sets of moderators, including clinical characteristics (e.g., anxiety disorders at baseline, anxiety severity, comorbid depression), previous treatments (psychotropic medication, psychotherapy) and sociodemographic characteristics (e.g., age group, sex, education level). Mediators will be examined for therapeutic alliance, group cohesion and adherence. Sensitivity analysis will be applied to examine the influence of missing data, and to document *per protocol* treatment effects (≥ 8 sessions completed).

##### Economic evaluation outcomes

The cost-effectiveness and cost-utility analysis will be carried out from health system and patient perspectives based on Canadian guidelines [77]. The 2-year costs considered will include all medical services and resources used during hospitalization, emergency department visits, outpatient visits, physician fees and outpatient medications. Patient out-of-pocket costs will include drug co-payments, payments to professionals not covered by the provincial public health insurance coverage, costs related to transportation and time spent by patients while seeking outpatient medical attention, as well as costs related to presenteeism and absenteeism [78]. Program costs associated with the training of facilitators and group meetings will include salaries, benefits, institutional overhead, and opportunity costs [79]. Generalized linear models (GLM) with log link and appropriate distribution (i.e., gamma) will be used to study the difference in costs (Beta estimates) as a function of the intervention (TAU vs SMS) while controlling for potential confounding study factors. Health outcomes will include symptom reduction (BAI) and health-related multi-attribute utility quality of life (AQoL-6D). The number of Anxiety-Free Days (AFD) will be calculated for each BAI scores with a value between 1 (‘anxiety free’) and 0 (‘fully symptomatic’) with linear interpolation to estimate the number of AFDs between baseline and follow up [80]. We will measure quality of life with the AQoL-6D to assess utility estimates [81]. For data analyses, repeated measures will be used to assess the difference (Beta estimates) in health outcomes as a function of the intervention and variables of interest. The incremental cost-effectiveness ratios (ICER) and incremental cost-utility ratios (ICUR) will be calculated based on beta estimates obtained. A discounting rate of 3.5% will be used for future values. We will carry out a sensitivity analysis for estimated values while considering a range of plausible values (95% CI). The ICUR will quantify the trade-off between costs and health-related quality of life.

### Trial coordination

The trial coordinating center will be at Université de Sherbrooke. The executive committee will be composed of the principal investigators, the principal knowledge user (or representative), and research coordinator, with web meetings every two to three weeks throughout the four years of the project, an efficient management strategy for multi-centric trials. A Steering committee (i.e., co-investigators and co-knowledge users) will meet at strategic decision-making points throughout the trial.

### Dissemination policy

We have adopted an integrated knowledge transfer (KT) strategy in which knowledge users are integral team members and participate in the complete research process. We have established a collaboration with the Relief community organization - involved in the design of the study, full members of the research team, and involved as knowledge users throughout all project phases. Other collaborators, including national, provincial, and regional decision makers, clinicians, and patient-partners for the advisory board, will also contribute specific expertise to integrated knowledge application. All knowledge users will be involved in making decisions concerning data collection, analyses, interpretation of results and knowledge transfer. The detailed KT plan for the study will include activities for the public, organizations/professionals, and scientific community. We will follow best practices recommended by scientific journals to determine authorship in publications.

### Monitoring Steering committee

The study will be overseen by an independent data and safety monitoring committee (DSMC) consisting of three members with collective expertise in statistics, health services research, and anxiety disorders. No interim analyses will be conducted. The Data Safety and Monitoring Committee (DSMC) could request allocation data for a specific participant in case of an incident. The DSMC will monitor patient recruitment, retention and adverse events using a prespecified adverse event reporting protocol. The DSMC will also conduct a semi-annual audit.

## Discussion

To our knowledge, this is the first randomized controlled trial to examine the effectiveness of a structured group SMS program specifically developed for anxiety disorders as a complement to usual care. The SMS intervention developed by Relief for anxiety disorders could promote personal recovery through psychoeducation, strategies for day-to-day symptoms management, self-efficacy and empowerment. In response to the knowledge gap about the added value of structured group SMS for anxiety disorders in community-based care, we have established a strong collaboration between researchers and knowledge users to address this question. This partnership will help us provide relevant data to knowledge users to increase the uptake of trial results. In Québec, this group-based SMS program for anxiety disorders developed and implemented by Relief has demonstrated good uptake, even without evidence-based data regarding the benefits of SMS as a complementary intervention for individuals with anxiety disorders. Moderator and mediator analysis will also provide informative hypothesis generating data for future trials to examine sociodemographic and clinical characteristics associated with SMS effectiveness, but also with regards to previous treatment experience. As there is an overlap between SMS and low-intensity psychotherapy interventions, this may provide interesting health services research hypothesis to inform future studies. Therefore, we will conduct this pragmatic randomized controlled trial to document potential benefits of SMS for individuals experiencing anxiety disorders with extensive patient-reported outcome measures as well as with health system data to inform policy makers, health care managers, clinicians, and patients on the potential impact of the intervention. The Relief community organization is committed to submitting the added value of the group SMS program to a rigorous evaluation, to share results to partner organizations as well as to review the methodology and content of the SMS intervention in case of negative results. A rigorous evaluation of the effectiveness and cost-effectiveness of SMS as a complement to treatment-as-usual could have a significant impact on evidence-based decision-making of stakeholders considering the upward emphasis on a personal recovery-based approach in mental health.

## Data Availability

The datasets generated during the current study will not be publicly available due to ethics committee regulations, but will be available from the corresponding author on reasonable request.

## Abbreviations

AFD: Anxiety-free days
AQol-6D: Assessment of Quality of Life – 6D
BAI: Beck Anxiety Inventory
BDCU: Quebec emergency department database
CBT: Cognitive behavioural therapy
CRCHUS: Centre de recherche du Centre Hospitalier Universitaire de Sherbrooke
DSM-5: Diagnostic and Statistical Manual of Mental Disorders, 5th edition
DSMC: Data and safety monitoring committee
GAD-7: Generalised Anxiety Disorder Questionnaire
GCS: Gross Cohesion Scale
GLM: Generalized linear models
ICER: Incremental cost-effectiveness ratios
ICUR: Incremental cost-utility ratios
KT: Knowledge transfer
MHSQ: Mental Health Self-Management Questionnaire
MINI: Mini International Neuropsychiatric Interview
PDSS-SR: Panic Disorder Severity Scale Self Report
PHQ-9: Patient Health Questionnaire
RAMQ: Régie de l’assurance-maladie du Québec
RAS-r: Recovery Assessment Scale – revised
RCT: Randomized controlled trial
SMS: Self-management support
SPIN: Social Phobia Inventory
TAU: Treatment-as-usual
WAI: Working Alliance Inventory
